# Climate predictors of child undernutrition: Insights from a machine learning model

**DOI:** 10.1371/journal.pone.0342448

**Published:** 2026-03-06

**Authors:** Fiifi Amoako Atta Panyin Essiam, Mary Amoako, Sameeratu Issah, Michael Biney, Kwadwo Owusu Akuffo, Leonard Kofitse Amekudzi

**Affiliations:** 1 Department of Biochemistry and Biotechnology, Faculty of Biosciences, College of Science, Kwame Nkrumah University of Science and Technology, Kumasi, Ghana; 2 Department of Meteorology and Climate Science, Faculty of Physical and Computational Sciences, College of Science, Kwame Nkrumah University of Science and Technology, Kumasi, Ghana; 3 Department of Optometry and Visual Science, Faculty of Biosciences, College of Science, Kwame Nkrumah University of Science and Technology, Kumasi, Ghana; Clinton Health Access Initiative, UNITED STATES OF AMERICA

## Abstract

**Background:**

Climate variability is increasingly recognized as a driver of child undernutrition, yet the non-linear relationships between specific climatic variables and nutrition remain unclear. This study uses machine learning to identify and quantify key climatic predictors of undernutrition among children.

**Methods:**

In a mixed-method approach, a cross-sectional study assessed nutrition and child health outcomes in May 2024, while retrospective climate data was assessed spanning January 2022 to December 2023. The cross-sectional study recruited two hundred and seventy (270) children aged 6–23 months from rural areas in the Bosomtwe district. Anthropometry, hemoglobin concentrations, and food frequency were assessed using standard procedures. The collected data was standardized and subjected to principal component analyses to identify dietary patterns. Household food security was assessed using the USDA Household Food Security questionnaire, while the climate data was obtained from ERA5 reanalysis. Random forest algorithms were employed to evaluate the relative importance of various climatic factors in predicting undernutrition and morbidity. Decision trees were then derived from the models to examine interactions and thresholds.

**Results:**

Rainfall emerged as the most critical climate predictors of stunting, while severe acute malnutrition was more sensitive to shortwave radiations. Temperature was the top predictor of fever, anaemia and diarrhoea. Low rainfall and high temperature substantially increased the possibility of undernutrition and morbidity. Threshold effects showed that rainfall below 4.78 mm and temperature under 23.40°C, increased stunting risk, especially when SW radiation drops below 6.01 W/m^2^. For severe acute malnutrition, rainfall below 5.53 mm and temperatures above 27.67°C significantly increased the risk.

**Conclusion:**

This study finds significant influences of climate variables on child undernutrition, highlighting the importance of integrated climate health strategies that account for compound climate effects. These findings can inform the development of early warning systems and targeted interventions to mitigate climate-related health risks in vulnerable populations.

## Introduction

Child undernutrition remains a significant public health concern globally [1], with the highest burden observed in low- and middle-income countries (LMICs) [2], particularly across sub-Saharan Africa [1]. Undernutrition contributes to nearly half of all deaths in children under five and is strongly associated with impaired physical growth, cognitive development and increased susceptibility to infectious diseases [3]. In Ghana, although progress has been made in reducing undernutrition [4], the prevalence remains unacceptably high in rural areas where poverty and food insecurity prevails [5]. Emerging evidence suggests that climate variability is an underappreciated yet potent driver of undernutrition [6,7]. Climate-induced disruptions such as variability in rainfall patterns, increased temperature and extreme weather events can affect food production, water availability [8] and disease transmission, thereby increasing children’s susceptibility to stunting, wasting and other forms of malnutrition [6]. In agrarian economies like Ghana, which rely heavily on rain-fed agriculture [9], variability in rainfall or temperature can destabilize household food security and health outcomes [10].

Despite growing recognition of the relationship between climate and nutrition, much of the existing literature relies on linear models that may under/oversimplify these relationships [11]. The complex, non-linear interactions among climate variables, and their thresholds require sophisticated tools for analysis. Recent advances in machine learning (ML) offer powerful tools to uncover complexities by capturing nonlinearities and high-dimensional interactions that traditional statistical methods may overlook [12]. Moreover, combining ML with explainable AI techniques enables the identification of critical thresholds and synergistic effects that can inform targeted interventions. Machine learning approaches such as random forests and decision trees offer a promising alternative to uncover hidden patterns that traditional statistical methods may overlook [13]. Our study also focuses on Ghanaian children, capturing local climate variability and its impact on undernutrition outcomes. Unlike previous studies that primarily assess single climate predictors, we applied machine learning methods, including Random Forest (RF) and Decision Trees (DT), to explore not only the most important predictors but also interactive effects and differing levels of climate variables on child malnutrition and cognition. This approach provides localized, data-driven insights that reflect Ghana’s unique climatic patterns, dietary behaviours and health environment. In light of these, this study aimed to provide insights on the predictive power of some climate variables on undernutrition, cognition and health outcomes among children 6–23 months in rural Ghana. We hypothesize that climate variables such as temperature and rainfall are significant predictors of undernutrition, cognition and health outcomes in this population. We offer a fresh data-driven viewpoint on how climatic exposures interact to influence child health by utilizing RF and DT.

## Methods

### Study design,setting and population

This study utilized a mixed-method approach to examine the relationship between climate indicators and nutritional outcomes. A cross-sectional survey was conducted to assess outcome variables (nutritional status, food security, food frequency and blood hemoglobin concentrations) in May 2024. This study focused on children aged 6–23 months. To examine potential climate-related influences on child nutrition, retrospective weather data spanning January 2022 to December 2023 were taken. This timeframe was selected to account for prenatal and early-life environmental exposures as the study cohort, born between June 2022 and November 2023, could have been affected by climate variability during both gestation and postnatal period. The study was conducted in the Bosomtwe district, a predominantly rural farming community in the heart of the Ashanti region of Ghana. The average household size in the district is four people per household, with about 22,895 households in total. It occupies a land size of about 422.5km^2^. Bosomtwe’s economy is dominated by agriculture, with over 62.9% of the labour force engaged in farming. Further, Bosomtwe is in the equatorial zone and experiences a rainfall pattern typical of the moist semideciduous forest region of the country. The area has two distinct rainy seasons, from March to July and September to November, while the dry season spans from December to March. The temperature remains consistently high, with an average of about 24 °C [14].

### Sampling strategy & recruitment

Before data collection, enumerators were trained on the survey instruments and child anthropometry. Enumerators who exhibited competence in the various tools participated in the data collection process. This ensured consistency and accuracy in the data collection process. This training equipped them with the necessary skills required to collect reliable information while adhering to ethical guidelines.

In each community, announcements were made to invite participants. All those interested in the study converged at the Community Health Post (CHPs) compounds and were taken through the voluntary consenting process. Participants who agreed to participate either thumb-printed or signed a consent form before the data collection processes began. Data was gathered using a semi-structured questionnaire. The questionnaire was designed to capture information about sociodemographic characteristics, anthropometry, food security and dietary assessment.

The sample size was determined using Cochran’s formula. A prevalence of 19% was used based on prior data from the Ghana Demographic and Health Survey [15]. A z-value of 1.96 which corresponds to a 95% confidence interval and a margin of error of 5% was used. Two hundred and seventy (270) infants aged 6–23 months living in 9 communities in the Bosomtwe district were enrolled. The communities included Sawua, Brodekwano, Kokodie, Apinkra, Akokofe, Kuntenase, Aputuogya, Nkwanta and Adumam.

## Data collection procedures

### Hemoglobin and malaria assessment

A urit meter (URIT-12, model number: 12Z278040) was used to determine hemoglobin concentration. The malaria status was determined using an antigen-based malaria rapid diagnostic test (RDT) kit (Hightop One Step Rapid Test, model number: H156). These tests were conducted using drops of blood obtained by puncturing the middle finger using a sterile single-use lancet.

### Morbidity

Morbidity was assessed using a questionnaire adopted from Rahman and Hossain [16]. The following question was asked to the mother of the child “Did your child suffered from diarrhea, fever, and ARI in the last 2 weeks?”. The answers were recorded as yes or no.

### Food security and dietary assessment

Food frequency questionnaire was assessed using a validated food frequency questionnaire comprising of 15 food items [17]. The collected data were standardized and subjected to Principal component analysis to identify dietary patterns. Household food security was assessed using USDA 18-item food security questionnaire [18].

### Climate data source

The ERA5 dataset, a reanalysis data from the the European Centre for Medium-Range Weather Forecasts (ECMWF) was used for this study. The data is high-resolution gridded global climate data at a spatial resolution of 0.25 x 0.25 [19]. For this study, a two-year subset (January 2022 to December 2023) was extracted and used climatic exposures for the study domain, because there were no meteorological stations within the study area. However, the ERA5 data has been validated over the region. This was done to capture the lagged effects of environmental exposures on child nutritional status. Existing literature supports that climatic factor such as rainfall variability, temperature extremes and radiation have cumulative impacts on food production, infectious disease exposure and household food security [20–22]. These factors also affect child growth outcomes over time [23,24]. Since anthropometric indices like height-for-age and weight-for-age reflect chronic and acute malnutrition status respectively, it is appropriate to assess climatic exposures over the preceding 12–24 months. This window aligns with the developmental period relevant to the children sampled in May 2024 and improves ecological validity of the model.

#### Child cognitive development.

The Caregiver Reported Early Development Instrument (CREDI) developed by the Havard University’s Graduate School of Education was used to assess early cognitive development [25,26]. Caregivers completed a long-form CREDI questionnaire designed to capture cognitive development skills. A point was awarded for each milestone they have achieved according to their age, if the child had not reached the milestone or the caregiver was uncertain, they received a score of 0. Norm-referenced z-scores were calculated from these raw scores using the web-based CREDI Scoring Application.

### Ethical approval

The study adhered to the principles outlined by the Declaration of Helsinki. Ethical clearance was obtained from the Bosomtwe District Health Directorate, and further approval was granted by the Committee on Human Research, Publication and Ethics at Kwame Nkrumah University of Science and Technology, Kumasi, Ghana (CHRPE/AP/067/24). Caregivers who consented to participate provided written informed consent by signing a consent form.

### Data analysis

All analysis were conducted using Python 3.12.7 (packaged by Anaconda, Inc.). Descriptive statistics were used to summarize demographic characteristics. Principal Component Analysis (PCA) was applied to food frequency data to identify dietary patterns with results interpreted based on factor loadings after applying varimax orthogonal rotation method [16]. The dietary patterns were named after food types with factor loadings above 0.50. This multivariate technique reduces the dimension of correlated dietary variables into fewer uncorrelated components, allowing for a more interpretable structure of eating behaviours. For continuous outcomes, Z-scores were obtained for stunting (height-for-age z-score), wasting (weight-for-height z-score), underweight (weight-for-age z-scores) and severe acute malnutrition (mid-upper arm circumference z-scores). In addition, total food insecurity scores, cognitive development z-scores and factor scores for the three identified dietary patterns were all treated as continuous variables. Categorical indicators included binary indicators (yes/no) of health status such as fever, diarrhoea, malaria and anaemia. In the predictive modelling, both continuous and categorical outcomes were analysed using Random Forest (RF) models implemented via scikit-learn (v1.4.0) library. *RandomForestRegressor* was used for continuous outcomes. *RandomForestClassifier* was used for categorical outcomes. Data was randomly split into training (70%) and test (30%) sets. The training set allowed the model to learn patterns and relationships form the majority of the data, while the test set was used to evaluate the model’s generalization ability on unseen data. Feature importance was derived using Gini impurity reduction and subsequently normalized to percentages to facilitate interpretation. Decision trees were extracted and visualized from the RF models to further enhance interpretability and identify interactions between climate variables and how they affect the nutritional and health outcomes.

#### Model validation and hyperparameter tuning.

To ensure robustness and generalizability of the RF models, a 10-fold cross-validation procedure was implemented. Hyperparameter tuning was conducted using a grid search approach optimizing the number of trees, maximum depth, minimum samples per split and the number of features considered at each split. Model performance was assessed using the coefficient of determination (R^2^), mean squared error (MSE), and root mean square error (RMSE) for continuous outcomes, and accuracy, precision, recall and F1-score for categorical outcomes. Table 1 summarizes the optimized model parameters and corresponding performance metrics for all outcomes.

**Table 1:** Performance metrics of RF models.

Outcome	Model Type	R²	MSE	RMSE	Accuracy	Precision	Recall	F1-Score
**Stunting**	Random Forest Regressor	0.31	2.49	1.58	–	–	–	–
**Underweight**	Random Forest Regressor	0.29	1.46	1.21	–	–	–	–
**Wasting**	Random Forest Regressor	0.22	1.69	1.30	–	–	–	–
**Severe Acute Malnutrition (SAM)**	Random Forest Regressor	0.29	1.10	1.05	–	–	–	–
**Cognition**	Random Forest Regressor	0.35	0.66	0.81	–	–	–	–
**Food Insecurity**	Random Forest Regressor	0.23	43.57	6.60	–	–	–	–
**Plant-Based & Whole-Foods Pattern**	Random Forest Regressor	0.30	0.72	0.85	–	–	–	–
**Sweet Tooth Pattern**	Random Forest Regressor	0.22	0.79	0.89	–	–	–	–
**Formula & Supplemental Feeding Pattern**	Random Forest Regressor	0.27	0.73	0.85	–	–	–	–
**Fortified Milk-Based Dietary Pattern**	Random Forest Regressor	0.23	0.77	0.88	–	–	–	–
**Fever**	Random Forest Classifier	–	–	–	0.73	0.88	0.29	0.43
**Diarrhoea**	Random Forest Classifier	–	–	–	0.74	0.93	0.28	0.43
**Respiratory Infection**	Random Forest Classifier	–	–	–	0.87	0.93	0.78	0.85
**Malaria**	Random Forest Classifier	–	–	–	0.98	1.00	0.60	0.75
**Anaemia**	Random Forest Classifier	–	–	–	0.74	0.72	1.00	0.84

RF = Random forest; R^2^ = coefficient of determination; RMSE = root mean square error. Reported metrics represent full-data model performance following 10-fold cross validation and grid-search hyperparameter tuning. Continuous outcomes were modelled using Random Forest Regressors and categorical outcomes using Random Forest Classifiers. Dashes (-) indicate non-applicable metrics

## Results

### Dietary patterns of infants

**Fig 1:**
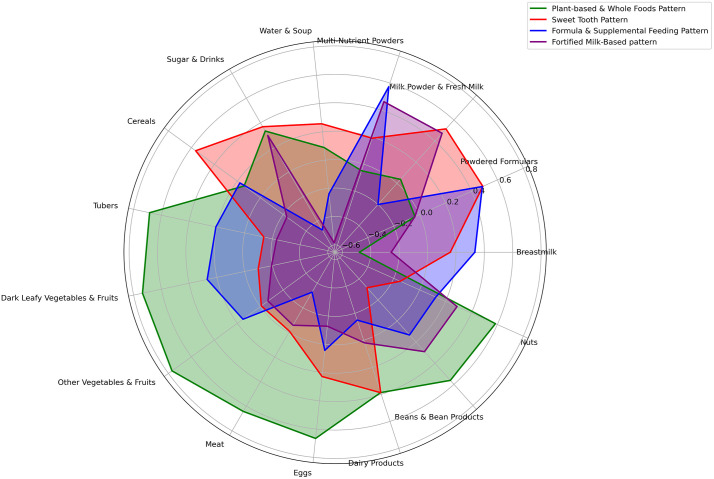
Dietary patterns of infants.

Fig 1 is a radar plot that shows the dietary patterns of the participants. Four major dietary patterns were identified; Plant based & Whole Foods (PBWF) Pattern, Sweet Tooth (ST) pattern, Formula & Supplemental Feeding (FSF) Pattern, Fortified Milk-Based (FMB) dietary pattern. The PBWF pattern was characterized by high factor loadings for tubers, cereals, vegetables, fruits and beans. The ST pattern was also defined by high levels of cereals, sugar, milk and milk products. The FSF pattern was characterized by high levels of powdered formula and multi-nutrients powders. Lastly, the FMB pattern was characterized by high levels of multi-nutrient powders and milk products.

### Model validation

RF regressors showed moderative predictive performance for continuous outcomes with R^2^ values ranging from 0.22 (wasting) to 0.35(cognition). RF classifiers also achieved high accuracy for outcomes ranging from 0.73 (fever) to 0.98 (malaria). F1-scores indicated a balanced performance, highest for respiratory infection (0.85) and malaria (0.75).

### Climate predictors of nutritional status, cognition, food insecurity and dietary patterns

**Fig 2:**
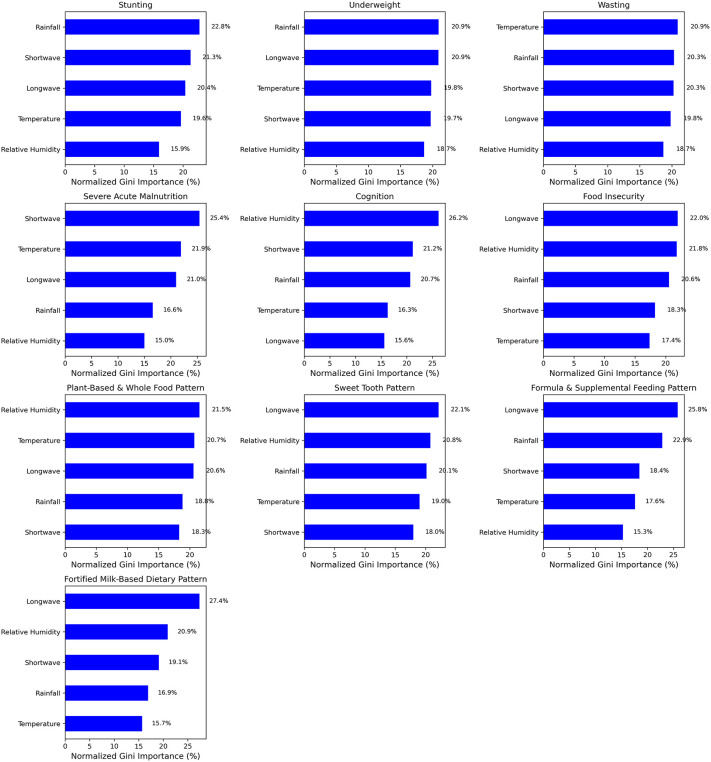
Predictors of Nutritional Status, Dietary Patterns and Cognition.

Rainfall (22.8%) and SW radiation (21.3%) emerged as the strongest predictors of stunting. Rainfall (20.9%) remained a dominant factor in predicting underweight. Temperature (20.9%) was strongly linked to wasting, followed by rainfall (20.3%). Severe acute malnutrition showed unique sensitivity to radiation variables, SW (25.4%) and LW radiations (21.9%) whiles food insecurity was strongly linked to LW (22.0%), relative humidity (21.8%) and rainfall (20.6%). Relative humidity (26.2%), SW radiation (21.2%) and rainfall (20.7%) were the top predictors of cognition. Regarding dietary patterns, the most important predictor of PBWF Pattern was relative humidity (21.5%), followed by temperature (20.7%), while long wave radiation was seen to predict ST pattern (22.1%), FSP pattern (25.8%) and FMB pattern (27.4%). These are shown in Fig 2.

### Climate predictors of morbidity

**Fig 3:**
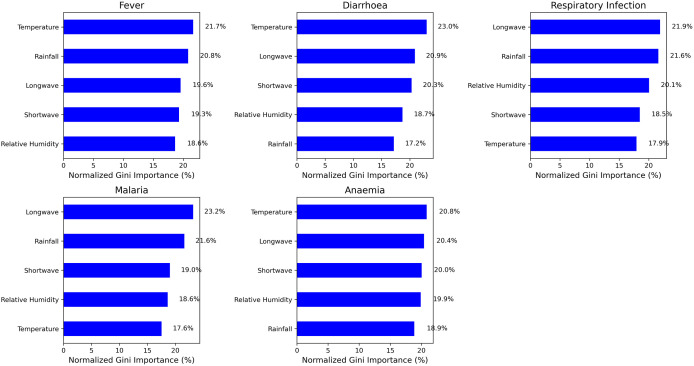
Climate predictors of infant morbidity.

Fig 3 shows the relative importance of climate variables in predicting morbidity outcomes. The model identified temperature (21.7%) and rainfall (20.8%) as the most dominant predictors of fever. LW radiation was the most important predictor for malaria (23.2%) and respiratory infection (21.9%) and the second most important predictor for anaemia (20.4%). Temperature (23.0%) and LW radiation (20.9%) and SW (20.3%) also emerged as the strongest predictors of diarrhoea.

### Hierarchical threshold interactions of climate drivers on malnutrition and cognitive development: Evidence from decision trees

**Fig 4:**
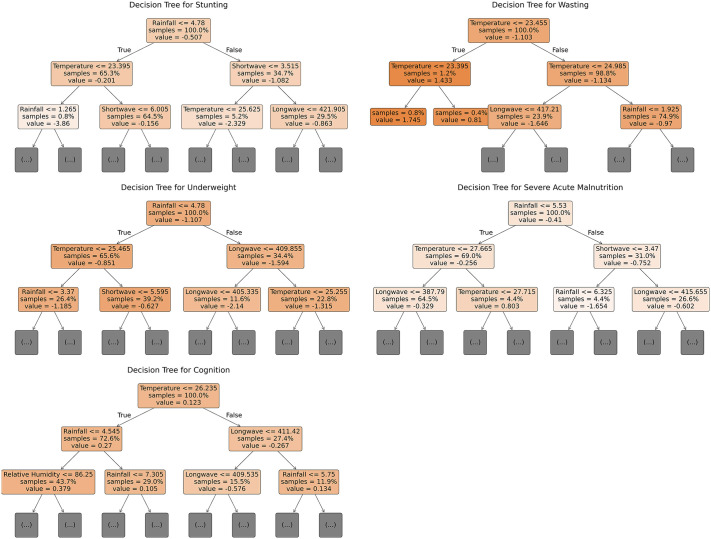
Decision tree showing interactions between climatic predictors of undernutrition and cognition.

The malnutrition and cognitive development indicators showed distinct hierarchical interactions between climate variables as shown by the decision tree splits in Fig 4. The root node split for stunting occurred at a rainfall threshold of 4.78 mm. Regions with rainfall ≤ 4.78 mm split into two child nodes; those with moderately cool temperatures (≤ 23.395 °C) exhibited improved stunting z-scores suggesting that moderate cooling synergizes with reduced rainfall to mitigate stunting severity. Regarding wasting, temperature emerged as the root node. Areas with moderate temperature specifically between 23.455 < T ≤ 24.985 °C together with LW < 417.21 W/m^2^ had lower wasting scores while those with lower temperature (≤ 23.395 °C) had better wasting scores. Rainfall emerged at the root node for underweight. Reduced rainfall (≤ 4.78 mm) together with low temperatures reduced the outcome of underweight. In areas with individuals exposed to higher rainfall and LW < 409.855 W/m^2^, underweight was higher. Low temperature (≤26.235 °C) together with rainfall below 5.545 mm contributed to better cognitive development. On the other hand, increasing temperature (> 26.235 °C) together with decreasing LW radiation (≤ 411.42 W/m^2^) contributed to worse cognitive development.

### Hierarchical threshold interaction of climate drivers on childhood morbidity: Evidence from decision trees

**Fig 5:**
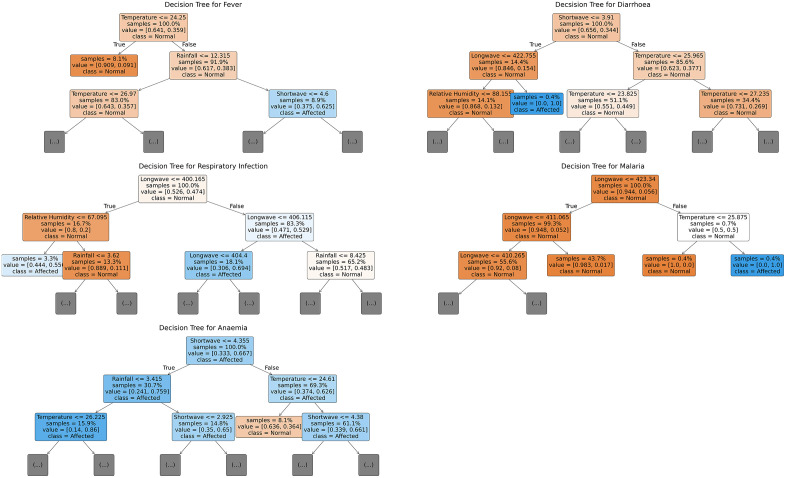
Decision tree showing interactions between climatic predictors of morbidity.

Decision tree analyses identified distinct climate variables as key predictors for childhood morbidity in Fig 5. The splitting determinant for fever was ambient temperature. Individuals exposed to temperatures ≤24.25°C were predominantly (62.7%) normal. Higher SW radiation (>4.6 W/m^2^) in conjunction with elevated temperature (> 24.25 °C) and rainfall (12.315 mm) was associated with a greater prevalence of being febrile (62.5%). SW radiation emerged as the initial splitting variable for diarrhoea. Lower exposures (≤3.91 W/m^2^) were associated with a 34.4% affected prevalence. Among those experiencing lower SW exposures, subsequent splits on LW and RH indicated that, lower LW ≤ 422.755 W/m^2^ and RH decreased the prevalence (13.2%). LW radiation was the first predictor of respiratory infection. Lower LW (≤ 400.165 W/m^2^) was associated with a 47.4% prevalence of respiratory infections. Together with low RH (≤ 67.095%), prevalence of respiratory infection decreased to 20%. Lastly, these LW, RH exposures together with rainfall > 3.62 mm further decreased the prevalence to 11%. On the other hand, increasing LW (> 406.115 W/m^2^) further increased the prevalence of diarrhoea to 52.9%. LW was again the first splitting predictor of malaria. Prevalence of malaria when LW radiation ≤ 423.34 was 5.6%. When LW decreased to about 411.065 prevalence reduced to 5.2%. On the other hand, when long waves > 423.34 W/m^2^ and temperature was around 24.875 or less °C, prevalence of malaria was 50%.

## Discussion

This study aimed to investigate the predictive power of specific climate variables on undernutrition and health outcomes among children aged 6–23 months in rural Ghana. Temperature and rainfall were the most important predictors of stunting and wasting. SAM was strongly associated with SW and LW radiations. Additionally, LW, rainfall and temperature emerged as key predictors of fever, diarrhoea, and respiratory infections. Decision trees showed a strong interactive effect, showing that a combination of low rainfall and high temperatures significantly increased the risk of undernutrition and morbidity.

### Predictors of stunting, underweight, wasting and severe acute malnutrition

Stunting was most strongly predicted by rainfall and SW radiation. This finding aligns with the body of literature which shows that rainfall variability affects linear growth in children [7,27,28]. Rainfall directly affects crop yield and food availability [29]. Inadequate or erratic rainfall variability affects agricultural productivity, household food security, dietary quality and reduces food availability, which over time, can impair linear growth in children. In Ghana, where there is a dependence on rainfed agriculture, erratic or rainfall variability leads to crop failures and food shortages, increase malnutrition risk in vulnerable age groups [7]. Moreover, high SW radiation may increase evapotranspiration (depleting soil moisture), and surface dryness [30], stressing water availability and reducing the productivity of staple and nutrient rich crops. These constraints can lead to poor maternal diets during pregnancy and lower-quality complementary feeding, both key contributors to stunting. For underweight, rainfall remained dominant emphasizing its criticality in food production systems. This finding is supported by research from Burkina Faso [24], which reported that regions suffering from reduced rainfall or drought experience 20–34% reduction in dietary diversity.

Wasting was primarily influenced by temperature, followed closely by rainfall. This observation mirrors longitudinal data from Sub-Saharan Africa, where each 1 °C rise in temperature increased wasting odds by 11–15% in African children [31]. Wasting in this study was probably influenced by heat-induced metabolic stress, reduced appetite and pathogen proliferation in warmer condition [30]. Severe acute malnutrition (SAM) showed sensitivity to SW radiation primarily, followed by LW radiation. SW radiation contributes to high solar flux which impairs hydration and thermoregulation [32], while LW radiation may contribute to nighttime heat retention [33]. These environmental conditions can worsen food shortages [24], and disrupt child care practices both of which increase SAM risk in vulnerable children.

### Predictors of cognitive development, food insecurity and dietary patterns

Cognitive development in infants and young children showed a unique sensitivity to relative humidity, SW radiation and rainfall. This suggests an indirect but powerful influence of climatic stressors on neurodevelopment. High humidity and SW radiation reflects indirect heat-humidity synergies, which impair neurodevelopment through maternal stress and inflammation during pregnancy [31]. Food insecurity was best predicted by longwave radiation, relative humidity and rainfall. This indicated that both hydrological factors influence household access to food. LW radiation intensifies nocturnal heat retention, accelerating grain spoilage in storage [34]. High humidity also fosters aflatoxin contamination in staple crops, reducing edible yields [31].

Regarding dietary patterns, specific climate predictors were linked to different dietary behaviours. The Plant based & whole foods (PBWF) pattern was strongly influenced by relative humidity, followed closely by temperature. Humid conditions support post harvest storage such as, the preservation of fresh fruits, vegetables and legumes by reducing dehydration, and preventing evapotranspiration hence maintaining soil moisture, thereby enhancing availability and accessibility of plant-based foods. This aligns with broader evidence that suggests favourable microclimates promote diets rich in minimally processed, nutrient-dense foods [11,35]. LW radiation was the dominant predictor for less nutritious dietary patterns including Sweet Tooth (ST) pattern, Formula and supplemental feeding (FSF) and Fortified Milk-based (FMB) patterns. Elevated LW radiation corresponds to increased surface heat retention. Physiological responses to this heat stress may alter appetite regulation, increasing preference for energy dense or sweet foods as coping mechanisms [36]. Moreover, the surface heat retention caused by high LW radiation can degrade fresh food quality and availability, pushing populations towards processed, shelf-stable or formula-based foods [35,36]. These patterns reflect climate-driven shifts in dietary choices mediated by both environmental constraints and behavioural adaptations.

### Predictors of common childhood morbidities

The identification of temperature and rainfall as the most dominant predictors of fever is well aligned with existing epidemiological evidence. Fever in young children often reflects underlying infectious diseases such as malaria, respiratory tract infections or viral illness [37]. Temperature influences pathogen replication rates and vector activity, while rainfall affects the availability of breeding sites for disease vectors such as mosquitoes [38,39]. Together, these factors create environmental conditions conducive to the transmission of febrile diseases. LW radiation emerged as a strong predictor for respiratory infections, malaria and anaemia. LW radiation, which reflects Earth’s energy emitted back to the atmosphere, is often associated with prolonged periods of heat, especially nighttime warming [40]. Such thermal stress can increase the burden of respiratory infections by influencing air quality and humidity levels, thereby affecting pathogen survival and human susceptibility [41]. The strong association for malaria is consistent with the sensitivity of mosquito vectors to temperature and LW radiation [38]. For diarrhoeal diseases, temperature and SW radiation were identified as the most influential climate predictors. This relationship is consistent with studies showing that high temperatures increase the proliferation of diarrhoeagenic bacteria in food and water supplies [42]. Meanwhile SW radiation, which intensifies solar heat and surface temperature may further degrade food safety and water quality, particularly in settings lacking adequate refrigeration and sanitation infrastructure.

### Interactive climate drivers of malnutrition and cognitive development

The root split for stunting occurred at a low rainfall threshold of 4.78 mm. This emphasizes the critical role of water availability in chronic malnutrition [43]. Regions experiencing rainfall below this threshold showed further stratification by temperature, where moderate cooling was associated with improved stunting z-scores. This suggests that in regions with low rainfall, lower temperatures may mitigate the adverse effects of the low rainfall on child growth. Thus, drier conditions accompanied by cooler temperatures may create less hostile environment for growth, possibly by reducing heat stress and conserving soil moisture which supports food production and subsequently nutrition. These findings agree with evidence from studies indicating that drought and heat stress synergistically exacerbate stunting risk in vulnerable populations [7,44].

In the case of underweight, rainfall was at the root node. Regions with reduced rainfall combined with low temperatures was associated with better scores. However, when rainfall increased and LW radiation dropped, underweight levels rose. This interaction suggests that excess rainfall when coupled with limited solar energy is often indicative of persistent cloud cover and may hinder food production, increase pathogen exposure and suppress immune functions, all contributing to weight deficits in young children [23,45]. Wasting identified temperature as the root determinant. Regions with warmer temperatures combined with high LW radiations were associated with better wasting scores. This finding suggests that regions with stable climatic conditions may provide consistent food supply [46] and immune functioning [47].

Regarding cognition, the tree showed that low temperature combined with moderate rainfall contributed to better cognitive outcomes, while increasing temperature alongside decreasing LW radiation was associated with poorer cognition. This suggests that cooler and moderately dry conditions may support neurodevelopment, possibly by reducing heat-related stress and limiting exposure to climate-sensitive infections that impair cognitive function. Recent studies in disadvantaged communities demonstrated that exposure to extreme heat can disrupt learning and cognitive performances during early childhood [48]. Furthermore, a narrative review confirms that climate change through temperature extremes and altered rainfall patterns negatively impacts children’s cognitive and mental health by increasing exposure to infectious diseases and stressors [49].

### Interactive climate drivers of childhood morbidity

Regarding fever, individuals exposed to lower temperatures predominantly exhibited normal health status. Conversely, higher SW radiation levels in conjunction with elevated temperatures and rainfall increased the likelihood of fever. This association aligns with findings from a systematic review which indicates that elevated temperatures can exacerbate heat-related illness in children due to under-developed thermoregulatory systems at that age [50]. SW radiation was the initial splitting variable for diarrhoeal disease. Lower SW exposure was associated with a high prevalence of diarrhoeal incidence. Among those experiencing lower SW exposures, lower LW and decreased relative humidity exposures reduced the prevalence. These findings emphasize the complex role of solar radiation in modulating water quality and pathogen viability, where low solar radiation may reduce pathogen die-off. LW radiation was the first node of respiratory infections. Lower LW radiation was linked to a high prevalence of respiratory infection. The prevalence initially decreased with decreasing relative humidity and decreased further with increasing rainfall. These observations suggest that reduced LW radiation and lower humidity may limit airborne pathogen survival or transmission, while rainfall may reduce particulate matter and airborne allergens that cause such respiratory conditions [51,52]. Lastly, LW radiation was the initial splitting predictor for malaria risk. When LW radiation was low, malaria prevalence was low, decreasing slightly with further reductions in LW radiation. However, when LW radiation and temperature increased, malaria risk sharply increased to 50%. This agrees with the known sensitivity of Anopheles mosquitoes to temperature and microclimate conditions [53] influenced by LW radiation which affects breeding and parasite development sites [54]. The United nations highlighted in 2012 that climatic conditions such as temperature and humidity have profound effects on longevity of mosquitoes and the development of malaria parasites, thereby affecting transmission rates [55].

### Strengths and limitations

Conducting this study in the Bosomtwe district, an agrarian community provides valuable insights into how climate variability affects child nutrition in settings heavily reliant on rain-fed agriculture. Further, this is one of the few studies to employ innovative methods to quantify and elucidate the critical relationships between climate factors and child health. One of the key strengths of our study is the use of machine learning approaches, which allowed for robust detection of non-linear relationships and complex interactions among multiple climate variables. The decision tree analyses also offer valuable insights into threshold effects and synergistic interactions. Despite these strengths, the study’s observational nature restricts the ability to establish causal relationships. One key limitation of this study is in the use of ERA5 reanalyses data, which though comprehensive and globally available, is model-derived rather than directly observed, as such ERA5 may not capture highly localized weather variation or microclimatic extremes due to spatial resolution and data assimilation methods. Additionally, while the study incorporated retrospective climate exposures to capture pre- and postnatal environmental influences, the observed associations could be affected by unmeasured confounding variables. Factors such as maternal nutritional status, agricultural practices, household income fluctuations and socio-political shocks were not directly assessed but could influence both climate exposure and child nutritional studies. Another limitation of this study is the potential for residual confounding. Although Random Forest models effectively capture complex, non-linear relationships, they do not provide adjustment for unmeasured or latent confounding variables in the same way traditional regression models do. Future research integrating these contextual variables and employing a longitudinal approach could provide more robust evidence regarding these associations.

## Conclusion

This study finds critical non-linear associations of multiple climate variables on childhood morbidity, cognitive development, food security and dietary patterns. These findings emphasize that climate impacts on child health are multifaceted and context-dependent, with specific thresholds of climatic factors shaping risk profiles. The identification of key thresholds and interactive effects between climatic variables offers compelling evidence of non-linear climate-health relationships with implications for both public health and climate adaptation strategies. These findings not only reaffirm that infant nutritional and health outcomes are sensitive to environmental stressors but also demonstrate the utility of machine learning techniques to unravel the complexities in the predictor-outcome dynamics in this area of research. The results of this study emphasize the need for integration of climate-sensitive approaches into nutrition surveillance and policy frameworks, especially in areas that are heavily impacted by climate change.

Future research should explore longitudinal designs to confirm causal relationships between climate and nutrition linkages. Also, the incorporation of higher resolution satellite or ground-station data available could improve the precision of climate estimates and help address the limitations of ERA5-derived data. The findings of this study, especially the threshold values identified, can inform climate-health early warning systems to anticipate and respond to nutritional and health risks among infants, especially during climate extremes. The findings of this study could also be translated into district-level interventions such as preventive feeding programs and climate-resilient agricultural support. Further, integration of community-based participatory approaches to co-design locally acceptable adaptation strategies that support climate resilience. Studies to include maternal nutrition, water security, and agricultural productivity could offer a broader picture on the climate-nutrition linkages.
